# Imported Furuncular Myiasis in a Non-Endemic Setting: Two Case Reports of *Dermatobia hominis* Infection in Romania and a Review of Reports from Southeast and Eastern Europe

**DOI:** 10.3390/tropicalmed11050110

**Published:** 2026-04-22

**Authors:** Gianluca D’Amico, Carmen Costache, Calin Gherman, Ioana Cristina Ilea, Adriana Györke

**Affiliations:** 1 Department of Parasitology and Parasitic Diseases, University of Agricultural Sciences and Veterinary Medicine, 400372 Cluj-Napoca, Romania; gianluca.damico@usamvcluj.ro (G.D.); calin.gherman@usamvcluj.ro (C.G.); adriana.gyorke@usamvcluj.ro (A.G.); 2 Emergency Clinical County Hospital, 400000 Cluj-Napoca, Romania; 3 Department of Microbiology, “Iuliu Hatieganu” University of Medicine and Pharmacy, 400349 Cluj-Napoca, Romania; 4 Ilea Ioana Cristina PFA on behalf of University of Agricultural Sciences and Veterinary Medicine, 400383 Cluj-Napoca, Romania; cristina.ilea@med-communication.com

**Keywords:** furuncular myiasis, *Dermatobia hominis*, travel-associated infection, COI sequencing, case report, narrative review

## Abstract

Furuncular myiasis is rarely reported in Southeast/Eastern Europe and may be underrecognized or misdiagnosed in non-endemic settings. We described two imported furuncular myiasis cases diagnosed in Romania following travel to Peru and confirmed the etiologic agent by larval morphology and mitochondrial cytochrome c oxidase subunit I (COI) sequencing. We also conducted a narrative review of published case reports/series from Southeast/Eastern Europe (1900–2025) and summarized case characteristics. A previously healthy 31-year-old woman and 32-year-old man presented with painful furuncle-like lesions on the upper back near the shoulder and the posterolateral upper arm, respectively, associated with pruritus and a sensation of movement. Each lesion had a central punctum with intermittent air bubbles. Occlusion of the breathing pore with petroleum jelly facilitated mechanical extraction of one barrel-shaped larva per lesion. Microscopy showed features consistent with second-instar *Dermatobia hominis* larvae, and COI sequencing demonstrated 97.14–99.33% identity with reference *D. hominis* sequences. Literature review identified 25 travel-associated cases, with *D. hominis* involved mostly after travel to Central/South America. These cases highlight the value of travel history and key diagnostic clues for *D. hominis* myiasis in travelers that may enable timely diagnosis and minimally invasive management. Greater awareness and reporting are needed to better define epidemiology.

## 1. Introduction

In humans, furuncular myiasis is a skin condition most commonly caused by the larvae of *Dermatobia hominis* (Linnaeus, 1781; human botfly) and *Cordylobia anthropophaga* (Blanchard & Beranger-Feraud, 1872; tumbu fly); less frequently by *Cordylobia rodhaini* (Gedoelst, 1910; Lund’s fly), *Cuterebra* spp., *Wohlfahrtia vigil* (Walker, 1849; myiasis fly), and *W. opaca* (Coquillett, 1897; fox maggot); and, rarely, *Hypoderma* spp. [1,2,3]. *Dermatobia hominis* is found in Central and South America, while *C. anthropophaga* is found in Eastern and Western Africa [4]. *Hypoderma* spp. (cattle bot fly) are non-tropical species with an origin in the Northern hemisphere [3].

These taxa share subcutaneous larval development but differ in how eggs/larvae reach the host. *Dermatobia hominis* glues eggs to blood-feeding arthropods (often mosquitoes) that deliver first-instar larvae to the skin, where warmth triggers hatching and penetration [4]. By contrast, *C. anthropophaga* lays eggs on fecally contaminated soil or damp clothing/linens, and contact with warm skin induces hatching and larval penetration [5]. Similarly, *C. rodhaini* may deposit eggs on dry sand or clothing, with human infestation typically accidental [6]. *Cuterebra* spp. females usually oviposit directly or near the host, and larvae enter through natural orifices or skin wounds [4]. Unlike these oviparous flies, *W. vigil* and *W. opaca* are larviparous sarcophagids that deposit live first-instar larvae directly onto the host, producing furuncular lesions or migrating from the subdermis into other tissues [3,4]. Among *Hypoderma* spp., *H. bovis* and *H. lineatum* are most commonly involved in human hypodermosis, and transmission may occur either through oviposition on human hair or by contact of newly hatched larvae from infected animals with exposed skin [3]. Recognizing these species-specific transmission routes and life-cycle characteristics is essential for accurate diagnosis, larva extraction, and prevention counseling (e.g., heat-ironing clothes in endemic areas, avoiding contact with contaminated soil or damp laundry).

Traveling to tropical and subtropical regions where furuncular myiasis is prevalent poses a high risk of skin infestation. Therefore, it is crucial to identify this condition in individuals returning from these regions [7]. Some predisposing factors for furuncular myiasis include advanced age, poor hygiene, and low socioeconomic status [8]. The prevalence is higher among individuals at the extremes of age, mainly due to poor self-hygiene [9].

A narrative review of Western European data identified 36 cases of furuncular myiasis reported until 2020. Of these, 39% were caused by *Cordylobia* spp. and 36% by *D. hominis* in travelers returning from endemic areas, and 25% were indigenous cases caused by *Hypoderma* spp. [8]. An increase in furuncular myiasis cases is expected in Europe due to climate change and the migration of fly species from tropical and subtropical countries [8].

Because furuncular myiasis occurs sporadically in non-endemic regions, misdiagnosis, delayed diagnosis, and mismanagement can occur, especially when epidemiological data are limited and disease awareness is low [10]. It is essential for physicians to promptly recognize the symptoms of furuncular myiasis, accurately diagnose it, and provide appropriate treatment.

We conducted a literature review to provide an overview of reported cases of furuncular myiasis in Southeast and Eastern European countries. Additionally, we present two cases of furuncular myiasis caused by *D. hominis* larvae in a young Romanian couple who had traveled to Peru on vacation. These cases highlight the importance of increasing physicians’ awareness of the disease. We provide details on the disease presentation, diagnosis, differential diagnosis, and therapeutic management to aid in this effort.

## 2. Materials and Methods

### 2.1. Case Reports

A 31-year-old previously healthy woman and a 32-year-old previously healthy man presented to the ambulatory service of the Emergency Clinical County Hospital, Cluj-Napoca, Romania, with a history of painful insect bites followed by painful cutaneous swellings with surrounding erythema resembling abscesses. The lesions were on the woman’s upper back near the shoulder and on the man’s upper arm. At the time of the current clinical examination, the lesions enlarged, and a small opening appeared in the center. Subsequently, both patients were referred to the parasitology section of the laboratory for further evaluation.

Demographic and clinical patient data were extracted from the hospital chart.

The larvae extracted from the furunculous lesions were referred to the Department of Parasitology and Parasitic Diseases at the University of Agricultural Sciences and Veterinary Medicine, Cluj-Napoca, Romania, for morphological identification.

### 2.2. Parasite Identification

We performed morphological identification [11] and determined the *D. hominis* larval instar.

Genomic DNA was extracted with a commercial kit (Isolate II Genomic DNA Kit, Bioline, London, UK) from an isolated specimen using 250 mg of parasite tissue and following the manufacturer’s instructions. The 710 bp mitochondrial cytochrome c oxidase subunit I (COI) gene fragment was PCR-amplified using primer pairs LCO1490/HC02198 [12]. DNA primers for amplification of mitochondrial cytochrome c oxidase subunit I from diverse metazoan invertebrates were used [12]. The PCR was performed as described in Dumitrache et al. (2023) [13]. The 710 bp PCR amplicon was visualized by electrophoresis on a 1.5% agarose gel. Then, the PCR product was purified using FavorPrep GEL/PCR Purification Mini Kit (Favorgen Biotech Corp., Pingtung, Taiwan) and further sequenced (Macrogen Europe, Amsterdam, The Netherlands). The obtained sequence was compared with the available sequences in GenBank.

Phylogenetic analysis was performed using MEGA 11.0 software. The evolutionary history was inferred using the Neighbor-Joining method and the Tamura–Nei genetic distance model. The bootstrap consensus tree, inferred from 1000 replicates, was used to represent the evolutionary history of the taxa analyzed.

### 2.3. Literature Review

We performed a narrative literature review of PubMed, EMBASE, and Web of Science databases to identify published reports of furuncular myiasis in Southeast and Eastern European countries between 1900 and 2025. The following search terms were used: “furunculosis myiasis” OR “furoncular myiasis” OR “cutaneous myiasis” OR “dermatobiasis” OR “Dermatobia” OR “Cordylobia” OR “furuncular myasis”, combined with the names of predefined countries in the target region: “Romania” OR “Latvia” OR “Czech Republic” OR “Czechia” OR “Estonia” OR “Slovenia” OR “Croatia” OR “Montenegro” OR “Russia” OR “Russian Federation” OR “Slovakia” OR “Hungary” OR “Bulgaria” OR “Belarus” OR “Lithuania” OR “Bosnia” OR “Herzegovina” OR “Georgia” OR “Armenia” OR “Ukraine” OR “Poland” OR “Serbia” OR “Moldova” OR “Moldavia” OR “Albania” OR “North Macedonia” OR “Azerbaijan” OR “Albania” OR “Hungary” OR “Cyprus” OR “Greece” OR “Montenegro” OR “North Macedonia”. Records retrieved from the three databases were screened sequentially, and duplicate citations identified across databases were removed manually; the exact number of duplicate records was not recorded separately.

Titles and abstracts were screened by one author, and articles describing case reports, case series, or literature reviews of cutaneous or furuncular myiasis were selected for full-text screening. In addition, full texts were reviewed when eligibility was uncertain, particularly for articles containing broader terms such as “study of cutaneous myiasis” or when the abstract was unavailable. Articles were included in our review if they reported human cases of imported/travel-associated furuncular myiasis in one of the predefined countries. Articles were excluded if they involved non-human cases; countries outside the predefined region, wound or other non-furuncular forms of myiasis; non-case-based publications; or unavailable full texts when the abstract did not provide sufficient case-level information. Additional Google searches and snowballing of references from the included articles were conducted to identify further relevant reports not retrieved through the database search.

From the included case reports/series, we descriptively extracted and summarized the following variables: reporting country, patient characteristics (number of patients, age, sex), clinical presentation (anatomic location of the lesion[s]), etiologic agent (fly species), diagnostic confirmation method (categorized as clinical only, morphology, morphology + molecular testing, or histopathology ± molecular testing), and predisposing exposure/risk factors. Data were descriptively analyzed.

## 3. Results

### 3.1. Case Report Description

Anamnesis revealed that both patients had traveled to Peru (South America) approximately one week before presenting to the ambulatory service. During the trip, they hiked through the forest areas of the Peruvian Amazon basin, a hot, humid tropical region with dense rainforest, abundant rainfall, and extensive river systems. They reported insect bites that gradually enlarged over time.

**Figure 1:**
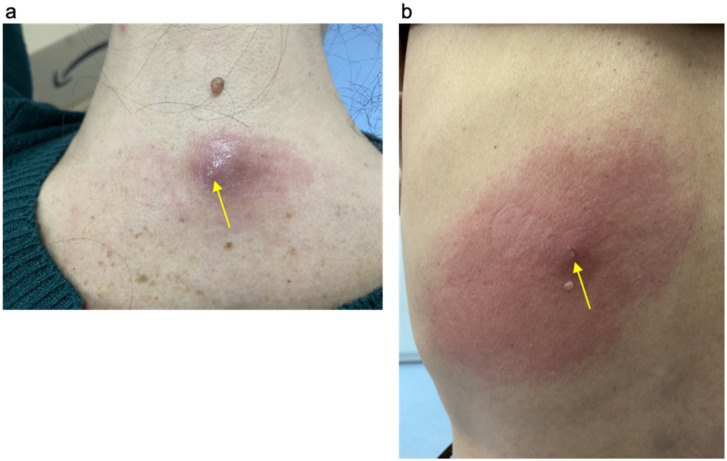
Stage II furuncular myiasis (B87.0 WHO ICD10-classification 2019) [14,15]; disease staging as proposed by Ragi SD, 2021 [16] in travelers returning from Peru: (**a**) furunculous-like lesions with reactive erythema and swelling located on the upper back near the shoulder in the woman; (**b**) furunculous lesions with reactive erythema and swelling located on the posterolateral upper arm near the deltoid/upper triceps region in the man. The arrows indicate the presence of tender erythematous nodules that contain larvae of *Dermatobia hominis*.

Physical examination revealed ~3 cm furunculous lesions with reactive erythema and swelling. The lesion was located on the upper back near the shoulder in the woman (Figure 1a) and on the posterolateral upper arm near the deltoid/upper triceps region, just below the shoulder in the man (Figure 1b). Small air bubbles were visible at the center of each lesion. Both patients reported a sensation of movement and itching at the lesion sites. Based on the clinical appearance and symptom duration, the presentation was consistent with furuncular myiasis (as defined by B87.0 WHO ICD10 classification 2019 [14,15]) of stage II (3–8 days) according to the staging proposed by Ragi SD et al., 2021 [16]. Both patients were otherwise healthy, had no systemic symptoms or known comorbidities, and reported no previous history of similar lesions or other known parasitic infections.

Considering the lesion characteristics, recent travel history, and antecedent mosquito bites, *D. hominis* infection was suspected.

Treatment involved mechanical removal of the larvae by applying a thick layer of petroleum jelly (Vaseline) over the breathing pore to induce localized hypoxia and encourage the larva to migrate toward the skin surface, making it easier to squeeze out [17]. To reduce the risk of secondary bacterial infection and support healing, we recommended a topical antibiotic ointment containing zinc bacitracin (250 IU/g) and neomycin sulfate (5000 IU/g) for at least 7 days. We also recommended regular wound cleansing and dressing changes until complete healing. The patients did not return for follow-up.

### 3.2. Parasite Identification Outcomes

**Figure 2:**
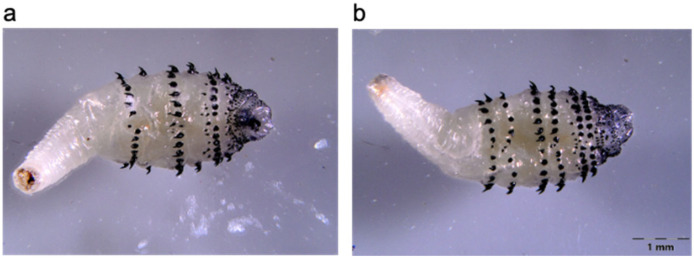
Second-instar larva of *Dermatobia hominis*: (**a**) ventral view; (**b**) dorsal view (Carl Zeiss microscope, Jena, Germany; x5 magnification).

One barrel-shaped larva was recovered from each lesion. The larvae were white-colored and measured approximately 10.5 mm in length and 4.5 mm in width (Figure 2). On microscopic examination, they showed a pyriform body shape, with an ovate to globular, spined anterior portion and a narrow, attenuated posterior portion. In addition, the thoracic and most abdominal segments bore heavily sclerotized, predominantly posteriorly directed spines, whereas the terminal abdominal segments had only delicate forward-pointing spines. Each larva also showed two spiracular slits in each posterior spiracular plate. These morphological features are consistent with a second-instar larva of *D. hominis* [11,18].

Morphological identification was further confirmed by sequencing the PCR product obtained from amplification of a 710 bp fragment of the mitochondrial cytochrome c oxidase subunit I (COI) gene. BLAST (NCBI BLAST, web version; National Center for Biotechnology Information, Bethesda, MD, USA) analysis of the resulting sequence showed between 97.14 and 99.33% nucleotide identity with *D. hominis* from Brazil (99.00–99.33%; GenBank accession nos. JQ246701.1, NC_006378.1, MT364820.1), Bolivia (98.83–99.17%; MG968179.1), Mexico (99.16%; MT159662.1), and Peru (97.14–97.39%; MT364820.1). The sequences were submitted to GenBank under accession numbers PZ185451 and PZ185506.

**Figure 3:**
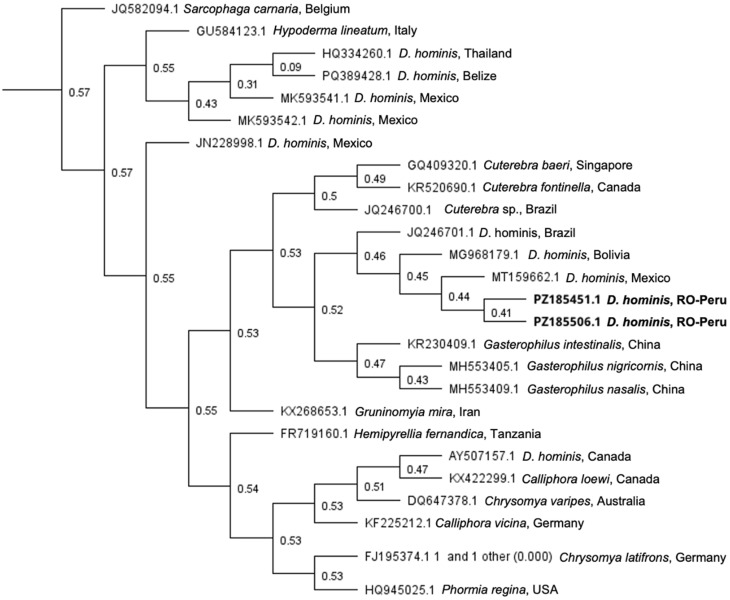
Neighbor-Joining tree based on mitochondrial cytochrome c oxidase subunit I (COI) gene sequences of the fly species causing myiasis. Notes: The two sequences obtained in this study are shown in bold. Numbers at the nodes indicate bootstrap support values. Reference sequences were obtained from GenBank.

Neighbor-Joining analysis of mitochondrial COI sequences showed that the two sequences obtained in this study clustered together within the *Dermatobia hominis* clade. The Romanian-Peru sequences grouped most closely with previously reported *D. hominis* sequences from Mexico (MT159662.1) and, more broadly, with isolates from Bolivia (MG968179.1) and Brazil (JQ246701.1) (Figure 3). Because branch support values within this subgroup were modest, the phylogenetic analysis is more informative for species confirmation than for detailed geographic inference.

### 3.3. Literature Review Outcomes

**Figure 4:**
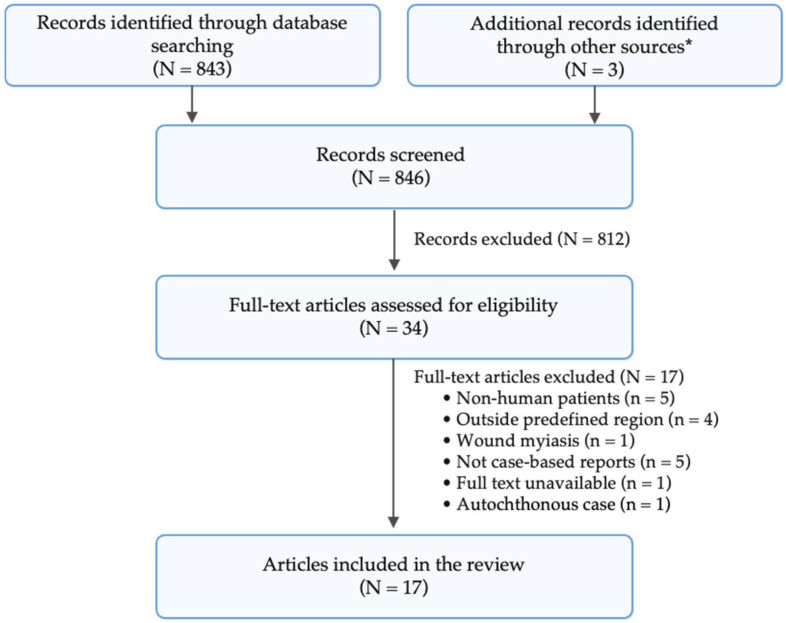
Flow diagram of the literature review. N, total number of articles; n, number of articles in a given category. * Google search and snowballing of the references included in the review.

Our database search yielded 843 articles on cutaneous furuncular myiasis. Duplicate citations identified across databases were removed manually during screening. Title and abstract screening yielded 31 articles for full-text review (29 indexed in PubMed and two from the local literature), of which 16 were excluded. Reasons for exclusion are shown in Figure 4. An additional Google search identified one extra article in the local literature reporting furuncular myiasis. Citation snowballing of the references cited in the included studies yielded two additional potentially relevant articles, of which one met the inclusion criteria and was included in the review (Figure 4).

In total, 17 articles describing 25 patients from Southeast and Eastern European countries were included in this narrative review. Patient age was available for 21 of 25 cases and ranged from 5 to 77 years; most reported patients were adults, with one pediatric case (5-year-old boy). Sex was reported in 21 cases (13 females, 8 males). Travel destinations were reported as Central and South America in 11 of 25 cases and sub-Saharan Africa in 11 of 25 cases. One case followed travel to Ladakh, northern India, and one older Polish report listed only “sailor” without destination. The most frequently identified agents were *D. hominis* (10 of 25 cases), associated with travel to Central and South America destinations. *Cordylobia* spp. accounted for 8 of 20 cases, including *C. anthropophaga* (n = 6) and *C. rodhaini* (n = 2), all associated with travel to sub-Saharan Africa destinations. One imported case was attributed to *Hypoderma lineatum* after travel to the Himalayan region. One Romanian case had disputed/uncertain species attribution. Four cases were classified based on clinical presentation and presence of larva, with *D. hominis* considered most likely in one patient and *C. anthropophaga* in three patients according to travel history and epidemiologic context. No reports have been identified in Albania, Armenia, Azerbaijan, Belarus, Bosnia and Herzegovina, Croatia, Cyprus, Estonia, Georgia, Greece, Hungary, Latvia, Lithuania, Moldova, Montenegro, Russia, and Ukraine.

Lesion location was reported for 21 of 25 cases. Lesions most commonly involved the lower extremity (n = 9) and trunk (n = 6), followed by the upper extremity (n = 4), gluteal region (n = 2), head/face (n = 1), and neck (n = 1); several cases had multiple lesions at different sites. Diagnostic confirmation was predominantly based on larval extraction with morphologic identification (n = 15). Molecular confirmation by COI sequencing was reported only in five cases with *Cordylobia* spp. identification.

Table 1 summarizes key patient characteristics and predisposing risk factors.

## 4. Discussion

Our literature review suggests that cutaneous furuncular myiasis is rarely reported in Southeast and Eastern European countries. Similar to other European settings, cases may be missed or misdiagnosed (e.g., sebaceous cysts or furunculosis) [34]. Although the clinical presentation of imported furuncular myiasis caused by *D. hominis* is well described in the literature, the present cases add regional documentation from non-endemic settings, and represent the first human cases in Romania and Southeast and Eastern Europe with species confirmation by mitochondrial COI sequencing. These data may contribute to future epidemiologic comparisons.

To date, in Romania, a potential *D. hominis* infection was first documented in 1994 in a 54-year-old woman who had traveled to California [24]. However, since *D. hominis* is not endemic in California, this report may have misidentified the causative (most plausibly *Cuterebra* spp.) or the origin of infection [35]. In 2023, a confirmed case of furuncular myiasis caused by *D. hominis* was reported in a 54-year-old man returning from a business trip to Brazil [25], and in 2025, a case was described in a 77-year-old man following travel to Brazil/Argentina [26]. In the latter report, species-level identification was not performed, but *D. hominis* was considered the most likely agent based on clinical features and travel epidemiology [26]. We now report two confirmed cases in a couple who returned from Peru. To the best of our knowledge, the present cases are the first in the country to have species confirmed by mitochondrial COI sequencing, in addition to morphological identification. Previously, *D. hominis* has been molecularly identified by COI sequencing only in an imported dog in Romania [35].

The travel history of our patients is consistent with other published case reports of furuncular myiasis caused by *D. hominis*, of which 10 (Table 1) occurred after returning from Peru, Bolivia, Chile, Guatemala, Honduras, El Salvador, Colombia, Brazil, Argentina, or Paraguay. This suggests that travelers to Central and South America remain at risk of *D. hominis* infection [36]. It is noteworthy that no cases have been reported in travelers returning from Mexico. Although *D. hominis* is distributed from Mexico to Argentina, traveler-related infections are most commonly reported from Belize, Bolivia, and Brazil. In Mexico, documented endemic areas are mainly in the southern region, particularly Yucatán and Quintana Roo [37,38]. The lack of published cases in Southeast and Eastern European travelers may reflect the geographically localized endemicity of exposure, relatively limited travel volume compared with other destinations, and possible under-recognition or misdiagnosis.

Reported cases in Romania appear to have increased over the past couple of years, potentially reflecting greater travel to some endemic tropical regions. Recent reports have also been identified in Poland, Serbia, Slovenia, and the Republic of North Macedonia. Of 10 reported cases, 9 were associated with *Cordylobia* spp. infection after travel to sub-Saharan Africa (Kenya, Gambia, Senegal, Uganda, Tanzania-Zanzibar), and 1 was associated with *D. hominis* infection after travel to Colombia [5,29,30,32,33]. Beyond travel, this apparent increase in reported traveler-associated cases may also be due to historically sparse epidemiologic data, previous underdiagnosis in non-endemic areas, currently improved clinical recognition, and more consistent reporting.

Data from Western Europe suggest that the incidence of cutaneous myiasis may rise in the coming years as warming climates expand the ecological suitability of myiasis-causing flies and as human mobility continues to introduce cases from tropical and subtropical areas [8]. In Romania, significant warming has been documented from 1961 to 2021, with increases in hot days and tropical nights, particularly in the southern, south-eastern, and western regions. Projections suggest that warming will continue, with August temperatures rising by more than 3 °C under the pessimistic scenario, while summer precipitation is expected to decrease in some regions [39]. These changes could theoretically increase environmental permissiveness for some thermophilic myiasis-causing flies, but direct evidence is lacking. In Europe, only 1 autochthonous case of furuncular myiasis caused by *D. hominis* has been reported in Portugal [40], suggesting that the event is biologically possible, but exceptional. *D. hominis* transmission depends on phoretic blood-feeding arthropods, commonly mosquitoes [4]. Although some of the implicated genera are present in Romania (e.g., *Aedes*, *Culex*) [41], this alone does not support the potential for sustained local transmission. Successful emergence of adults, mating, suitable humidity/temperature, access to phoretic carriers and vertebrate hosts, and successful pupation in the environment would also be required. Therefore, *D. hominis* infections diagnosed in Romania should still be regarded primarily as travel-associated imported infections.

Accurate diagnosis, larval identification, and systematic case reporting are important for better defining epidemiologic patterns and raising awareness among clinicians [8], particularly in non-endemic settings where unfamiliar presentations can delay recognition and lead to inappropriate management.

In our two patients, the diagnosis was guided by a combination of key clinical clues: travel to an endemic region (Peru) within the preceding week, an antecedent insect bite during travel followed by a gradually enlarging, painful furuncle-like lesion, and a central punctum with intermittent “air bubbles”. Both patients also reported pruritus and a sensation of movement at the lesion site. Overall presentation in our patients was consistent with the classical dermatologic and clinical descriptions of *D. hominis* furuncular myiasis provided by Maier and Hönigsmann (2004) [42] and by Mahal and Sperling (2012) [43], particularly with regard to recent travel to an endemic region, antecedent insect bite, and the presence of a furuncle-like lesion with a central punctum and local discomfort/pruritus. Based on symptom duration and lesion morphology, the presentation was consistent with stage II (furuncular) myiasis (3–8 days) as described by Ragi et al. (2021) [16].

Lesions often occur on exposed areas (e.g., back, shoulders, extremities), as observed in our two patients and in those identified in the literature (Table 1), and may become apparent weeks after the initial mosquito bite [7,16]. Ragi et al. proposed a clinical staging system based on the developmental progression of *D. hominis* larvae in the skin, which pass through three instars. Stage I or papular (0–2 days) is characterized by a “papule or nodule resembling an insect bite with diffuse itchiness” [16]; the first instar resembles a worm-like white structure with a bulbous end [18]. Stage II or furuncular (3–8 days) is characterized by a “larger, boil-like nodule with a central punctum (larval air hole) and surrounding erythema, discharge, and/or tenderness and pain” [16]; the second-instar larva has a bottle-neck shape [18]. Stage III or protuberant (9–12 days) is marked by protrusion of a fully developed “third-instar larva, cylinder-shaped, exiting the host to enter the ground to pupate” [16,18]. Each instar possesses backward-projecting black spines that encircle the thorax [18].

Dermatologic examination can help visualize parts of a *D. hominis* maggot within the nodule, while ultrasound or high-frequency probes can confirm the presence of a larva [16]. Differential diagnosis includes folliculitis, furuncle, epidermal cyst, or an embedded foreign body with secondary impetigo, and other parasitic infestations such as bedbugs, tungiasis, *C. anthropophaga*, *Hypoderma* spp., *Cochliomyia hominivorax*, etc. [16]. Species identification in our patients was confirmed by mitochondrial COI sequencing. The Romanian-Peru sequences clustered within the *D. hominis* clade and grouped most closely with a previously reported Mexican sequence, and more broadly with Bolivian and Brazilian isolates, supporting species-level identification. However, because branch support values within this subgroup were modest, the analysis is better interpreted as confirmation of taxonomic assignment than as evidence for detailed geographic relationships. This complements the previous study by Toussaint-Caire et al. (2018) [44], who showed with ITS-2 that molecular analysis can support species identification, and by Lozano-Sardaneta et al. (2020), who demonstrated the utility of COI for rapid molecular confirmation of *D. hominis* [45].

Furuncular myiasis can be managed by occluding the larval respiratory spiracles with substances such as petroleum jelly (Vaseline), clear nail polish, pork fat, tobacco tar, raw beef, or bacon strips [46]. When the larva protrudes to reach air, it can be gently removed; intact extraction is preferred, as larval fragmentation may prolong inflammation and necessitate surgical removal. If occlusive methods lead to larval asphyxiation and fragmentation, or if diagnostic uncertainty or significant secondary infection is suspected, surgical or vacuum extraction may be required. Ultrasound may be helpful in equivocal cases or when retained larval material is suspected. After removal, the affected area should be cleaned, secondary bacterial infection treated, and tetanus prophylaxis considered [46]. Anecdotal reports suggest ivermectin or tiabendazole as pharmacologic options, but controlled clinical data are lacking [8]. In our patients, occlusion with petroleum jelly followed by mechanical extraction was successful, and local wound care and topical antibiotic ointment were recommended. Both patients reported symptomatic relief after extraction, but they did not return for scheduled follow-up, precluding formal assessment of time to complete healing or recurrence. These cases underscore that early recognition of the characteristic lesions and travel history can avoid unnecessary incision and drainage or antibiotic overuse.

Preventive counseling for travelers to endemic regions should be included in daily practice and should advise avoiding sleeping naked, outdoors, or on the floor, and using raised beds or cots in screened huts or tents whenever possible. Additional measures include wearing long-sleeved clothing and trousers, preferably treated with pyrethrin or pyrethroids, and applying insect repellent to exposed skin [46,47].

## 5. Conclusions

Furuncular myiasis remains an uncommon and likely underrecognized diagnosis in Southeast and Eastern Europe, where imported cases may be misdiagnosed as bacterial skin infections. We report two confirmed cases of *D. hominis* in a couple returning from Peru and, to our knowledge, provide the first Romanian cases with species confirmation by COI sequencing, in addition to morphologic identification. These cases underscore the value of travel history and key clinical clues (a furuncle-like lesion with a central punctum, pruritus, and a sensation of movement) in helping achieve timely diagnosis, minimize invasive extraction, and avoid unnecessary surgical procedures or antibiotic overuse.

Future efforts should focus on standardized case reporting and regional surveillance, with broader access to COI-based species confirmation, to better define the incidence, causative species, and clinical outcomes of imported furuncular myiasis in Southeast and Eastern Europe.

## Figures and Tables

**Table 1:** Cutaneous furuncular myiasis: case reports in Southeast and Eastern European countries (1900–2025).

Author	Year	Country	No. of Pts	Age (Years)	Sex	Lesion Location	Fly Species	Diagnostic Confirmation	Risk Factor
Logar et al. [19]	2001	Slovenia	1	25	M	Ankle	*D. hominis*	Morphology	Travel to Peru
Logar et al. [20]	2006	Slovenia	1	48	M	Back	*C. anthropophaga*	Morphology	Travel to Ghana
			1	47	F	Nose, shoulder, wrist	*C. anthropophaga*	Morphology	Travel to Ghana
			1	14	F	Back	*C. anthropophaga*	Morphology	Travel to Ghana
Logar et al. [21]	2008	Slovenia	1	47	F	Neck	*Hypoderma lineatum*	Morphology	Travel to Ladakh, Northern India (Himalayan area)
Bartenjev et al. [22]	2025	Slovenia	1	35	F	Ankle	*D. hominis*	Morphology	Travel to Colombia
Totkova et al. [23]	2016	Slovakia	1	58	F	Gluteus maximus muscle	*D. hominis*	Morphology	Travel to Central America
Olteanu et al. [24]	1994	Romania	1	54	F	Not reported	*Dermatobia or Cuterebra*	Disputed/uncertain species	Travel to California
Acăei et al. [25]	2023	Romania	1	54	M	Abdomen	*D. hominis*	Morphology	Trip to Brazil
Blănaru et al. [26]	2025	Romania	1	77	M	Upper limb	*D. hominis (most likely)*	Clinical only *	Travel to Brazil and Argentina
Hozáková et al. [10]	2016	Czech Republic	1	44	F	Calf	*D. hominis*	Morphology	Travel to Peru, Bolivia and Chile
			1	29	F	Lower leg	*D. hominis*	Morphology	Travel to Guatemala, Honduras and El Salvador
Wegner et al. [27]	1986	Poland	1	-	-	-	*-*	Unclear/not stated	Sailor
Waśniowski and Rehlis, 2006 [28]	2006	Poland	1	35	M	Trunk and lower leg	*D. hominis*	Morphology	Vacation in Ecuador and Peru
			1	45	F	Back between the shoulder blades	*C. rhodaini*	Morphology	Travel to Rwanda and Uganda
Biernat et al. [29]	2024	Poland	1	30	F	Thigh	*D. hominis*	Morphology	Trip to South America (Bolivia, Peru, Paraguay)
Biernat et al. [30]	2025	Poland	1	-	-	-	*C. rodhaini*	Morphology + molecular (COI)	Trip to Uganda
	2025	Poland	1	-	-	-	*C. anthropophaga*	Morphology + molecular (COI)	Trip to Gambia
	2025	Poland	1	-	-	-	*C. anthropophaga*	Morphology + molecular (COI)	Trip to Gambia and Senegal
Velev et al. [31]	2019	Bulgaria	1	5	M	Forearm	*D. hominis*	Morphology	Travel to Brazil
Dragonjić et al. [5]	2023	Serbia	1	44	M	Abdomen and hand	*C. anthropophaga*	Histopathology + molecular (COI)	Temporary work in Mombasa, Kenya
Momčilović et al. [32]	2025	Serbia	1	24	M	Thigh	*C. anthropophaga*	Morphology + molecular (COI)	Trip to Kenya
Jusufovski et al. [33]	2024	Republic of North Macedonia	1	12	F	Upper left arm, glutes, lower right arm	*C. anthropophaga (suspicion)*	Clinical only *	Vacation in Tanzania-Zanzibar
			1	35	F	Lower part of both legs	*C. anthropophaga (suspicion)*	Clinical only *	Vacation in Tanzania-Zanzibar
			1	58	F	Lower part of both legs	*C. anthropophaga (suspicion)*	Clinical only *	Vacation in Trip to Tanzania-Zanzibar

* species-level identification not performed; F, female; M, male; COI, cytochrome c oxidase subunit I gene.

## Data Availability

Data supporting this article are not publicly available, due to the inclusion of sensitive and confidential information, including patient data. De-identified data or other information may be available from the corresponding author upon request, but are subject to ethical and institutional approvals.

## Abbreviations

The following abbreviations are used in this manuscript:

bp	Base pair
COI	Cytochrome c oxidase subunit I
DNA	Deoxyribonucleic acid
F	Female
ICD-10	International Classification of Diseases, 10th Revision
IUs	International units
M	Male
PCR	Polymerase chain reaction
UK	United Kingdom
WHO	World Health Organization
